# Effects of Olive Oil Consumption on Cardiovascular Risk Factors in Patients with Fibromyalgia

**DOI:** 10.3390/nu12040918

**Published:** 2020-03-27

**Authors:** Alma Rus, Francisco Molina, María Josefa Martínez-Ramírez, María Encarnación Aguilar-Ferrándiz, Ramón Carmona, María Luisa del Moral

**Affiliations:** 1 Department of Cell Biology, University of Granada, Avenida de la Fuentenueva, s/n, 18071 Granada, Spain; mrus@ugr.es (A.R.); rcarmona@ugr.es (R.C.); 2 Instituto de Investigación Biosanitaria ibs.GRANADA, 18012 Granada, Spain; e_aguilar@ugr.es; 3 Department of Health Science, University of Jaén, Campus Las Lagunillas s/n, 23071 Jaén, Spain; mjmartin@ujaen.es; 4 Department of Physical Therapy, University of Granada, Avenida de la Ilustración, 60, 18016 Granada, Spain; 5 Department of Experimental Biology, University of Jaén, Campus Las Lagunillas s/n, 23071 Jaén, Spain; mlmoral@ujaen.es

**Keywords:** cortisol, erythrocyte sedimentation rate, fibrinogen, fibromyalgia, olive oil, platelet distribution width

## Abstract

We have recently reported that patients with fibromyalgia (FM) may be at increased risk for cardiovascular disease. Olive oil reportedly has cardioprotective effects. We examined the influence of olive oil consumption on cardiovascular risk factors in FM. This preliminary study was performed on blood samples of women with FM who consumed 50 mL of organic olive oil daily for 3 weeks. Patients were randomized into two groups: 15 women ingested extra virgin olive oil (EVOO) and 15 refined olive oil (ROO). Cardiovascular risk markers were measured at baseline (pre measure) and after consumption of olive oil (post measure). Red blood cell count and erythrocyte sedimentation rate (ESR; both *p* < 0.05) declined significantly post-treatment in the EVOO group. Consumption of ROO increased mean platelet volume and reduced platelet distribution width (PDW), neutrophil-to-lymphocyte ratio, ESR and fibrinogen (all *p* < 0.05). Significant differences were found in pre–post change between the EVOO and ROO groups for cortisol and PDW (both *p* < 0.05). Our results have shown that consumption of olive oil may have antithrombotic and antiinflammatory properties in patients with FM, thereby improving a number of cardiovascular risk markers. Both EVOO and ROO may be useful as adjuvants for the prevention and/or treatment of cardiovascular disorders in these patients.

## 1. Introduction

Fibromyalgia (FM) is a prevalent disorder that is characterized by widespread chronic pain. It is frequently associated with sleep problems, fatigue, other functional somatic symptoms, mental and physical disorders and diminished quality of life [1]. Mortality risk related to FM has been reported to be 30% greater than in patients without this syndrome. This risk is not due to the disease itself, but the lifestyle of patients with FM, including lack of exercise [2].

There is still controversy concerning the etiopathogenesis of FM, and several factors have been proposed to be related to this syndrome, such as central sensitization [3], inflammation [4] and oxidative stress [5]. Due to the unknown etiology of FM, there is no effective treatment available. The treatment of FM requires a multidimensional approach that involves physical, pharmacological and cognitive measures [6]. The most strongly recommended forms of treatment are small doses of tricyclic antidepressants, cardiovascular exercise, cognitive behavioral therapy and patient education [6]. 

We have recently reported a prothrombotic state in patients diagnosed with FM, reflected by altered levels of thrombosis-related parameters, such as fibrinogen; prothrombin time; red blood cell (RBC); and platelet counts, platelet distribution width (PDW), mean platelet volume (MPV) and platelet-to-lymphocyte ratio (PLR) [7]. This prothrombotic state may increase the risk for cardiovascular disease in patients with FM.

The Mediterranean diet has been associated with longevity and with reducing the risk for cardiovascular diseases, cancer, neurodegenerative diseases and diabetes mellitus [8]. The beneficial effects of the Mediterranean diet have been attributed to the consumption of olive oil as well as of vegetables, fruits, nuts, legumes, unprocessed cereals, and fish. Several studies have shown the cardioprotective, antithrombotic and antiinflammatory properties of olive oil [9,10,11]. There are two types of olive oil depending on the production mechanisms, extra virgin olive oil (EVOO) and refined olive oil (ROO). Both types of olive oil have the same composition in monounsaturated fatty acids (MUFAs), mainly oleic acid, but differ in the contents of minor components. About 98–99% of the total weight of EVOO is represented by MUFAs. Minor components, classified as unsaponifiable compounds (squalene, sitosterols, triterpenes, pigments, etc.) and hydrophilic compounds (α-tocopherol, phenolic compounds, etc.), represent the remaining 1–2% [8,10]. EVOO is obtained by physical processes; that is, by crushing and pressing the olives. Conversely, ROO is submitted to chemical extraction mechanisms, such as refinement, which eliminates the majority of the minor components [8]. The beneficial properties of olive oil, including the cardioprotective ones, have been long attributed to its high MUFAs content. However, several studies have also highlighted the antioxidant beneficial effects of the minor components (mainly phenolic compounds) of EVOO regarding reducing thrombotic risk, inflammation, atherosclerosis, oxidative stress and cancer [11,12,13]. Most previous studies made no distinction among the different varieties of olive oil (EVOO or ROO) when investigating the effects of olive oil consumption on health, despite the different contents of minor bioactive components.

It has been reported that olive oil, especially EVOO, has beneficial effects on cardiovascular risk factors, such as coagulation, platelet aggregation, lipids, endothelial function and inflammation [8]. To the best of our knowledge, there are no other studies examining the cardioprotective properties of olive oil in patients with FM. Therefore, the goal of the present study is to investigate the effects of two olive oils with different minor component contents on cardiovascular risk factors (blood-coagulation parameters, platelet indices, red blood cell count, inflammatory markers, lipid profile, nitric oxide (NO) and cortisol) in patients diagnosed with FM.

## 2. Materials and Methods 

### 2.1. Participants

Female patients diagnosed with FM from AFIXA (Association of Fibromyalgia of Jaén, Spain) participated in this preliminary study, and provided written informed consent. Only women were selected based on FM prevalence data by gender, which is higher in women than in men [14,15]. The patients had been previously diagnosed with FM by a professional rheumatologist and met the American College of Rheumatology (ACR) criteria for FM. Exclusion criteria included the presence of any other chronic disease (ischemic heart disease, hypertension, cancer, diabetes mellitus), pregnancy, lactation, digestive intolerance to olive oil and grade II obesity (body mass index (BMI) ≥ 35 kg/m2). Participants were not consuming any medication known to affect antioxidative status. They were not under the treatment of anticoagulants, corticosteroids, estrogens, analgesics or antiinflammatory drugs, and were only included if they had stopped using them at least two months before the study. None were consuming alcohol, and all of them were non-smokers. All the participants were sedentary living women. 

### 2.2. Determination of Sample Size 

Sample size was determined using Ene 3.0 software (GlaxoSmithKline, Rockbille, MA, USA). To achieve a power (1 – β error) of 0.08, taking into account a significance level (α error) of 0.05 and based on the results of fibrinogen levels from a previous study evaluating the effects of olive oil on healthy subjects [16], it is necessary to include 13 subjects per experimental group in the study. In order to account for midtrial withdrawals, we enlarged the experimental groups to 15 participants.

### 2.3. Study Design

**Figure 1:**
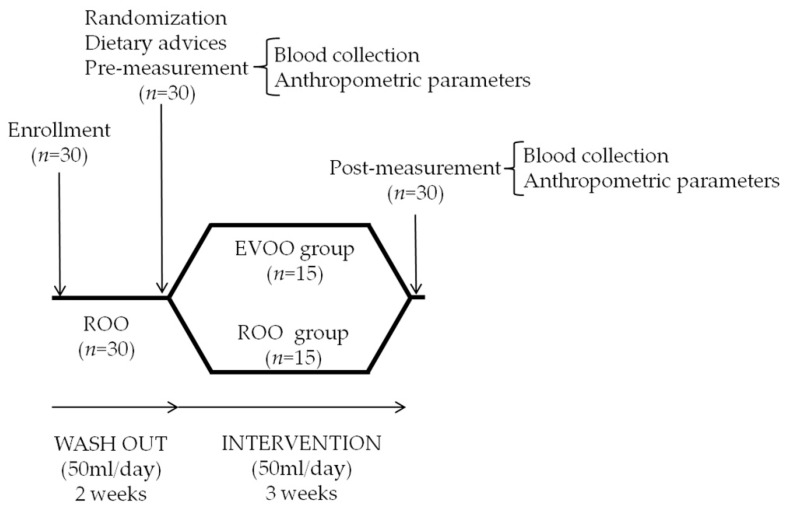
Study protocol. Note. EVOO: extra virgin olive oil; ROO: refined olive oil.

A randomized, controlled, double-blind nutritional trial was performed on 30 patients with FM. Trial registration number: NCT04245592. The research has been carried out in accordance with the Declaration of Helsinki [17] of the World Medical Association. The study was approved by the Ethics Committee of the University of Jaén (Spain). The trial consisted of consuming, for three weeks, two organic olive oils: extra virgin olive oil (EVOO) and refined olive oil (ROO). The composition of EVOO and ROO is presented in Table 1. Patients were divided into two groups: (I) EVOO group: 15 patients with FM consuming 50 mL/day of an organic EVOO; and (II) ROO group: 15 patients with FM consuming 50 mL/day of an organic ROO. Since the subjects were part of a population that consumes olive oil regularly, a washout period with ROO (50 mL daily, 2 weeks) was applied prior to the intake of EVOO and ROO. Blood markers and anthropometric parameters (weight, BMI and waist circumference) were measured in each patient both prior to and after the intake of olive oils. Organic olive oils were obtained from Olifarma S.L. (Granada, Spain). Both types of olive oil were similarly packaged and patients were blinded as to the olive oil they consumed. The olive oil was consumed raw. Participants used ROO for cooking in order to ensure their ingestion of antioxidants unchanged. We supplied ROO for cooking in sufficient quantity for the whole family. We estimated the participants’ intake by means of a 24 h recall that they completed for 3 days (2 working days and a day off) at the beginning of the trial. We used the averages of the values obtained over the 3 days to calculate each participant’s energy intake (kcals/day); intake of macronutrients (carbohydrates (g/day), lipids (g/day) and protein (g/day)); and intake of micronutrients, especially those that may have an antioxidant effect, including vitamin C, vitamin A, copper, zinc and selenium. Based on this analysis, we provided all participants with dietary recommendations for normalizing each participant’s intake of antioxidants during the trial. Patients with FM were requested to return all the olive oil containers (the consumed and unconsumed containers). As a control for the consumption of olive oil, a count of the containers returned by patients (the empty containers and the containers not consumed) was performed at the end of the nutritional trial. The study protocol is presented in Figure 1. 

### 2.4. Blood Collection and Preparation of Blood Samples

After participants fasted overnight, we drew venous blood in the early morning from the antecubital vein into two tubes with anticoagulant and two tubes without anticoagulant. The tubes with anticoagulant were centrifuged at 3500 rpm for 5 min at 4 °C to obtain plasma samples. Tubes without anticoagulant stood at room temperature for 30 min until the blood clotted, and were then centrifuged at 3500 rpm for 5 min at 4 °C to obtain serum samples. Blood was collected at the same time of the day to prevent daily variations in the levels of the laboratory parameters. Thrombosis-related parameters (fibrinogen levels, prothrombin time, cephaline time, platelet count, platelet distribution width (PDW), mean platelet volume (MPV), red blood cell (RBC) count, neutrophil-to-lymphocyte ratio (NLR) and platelet-to-lymphocyte ratio (PLR)) and erythrocyte sedimentation rate (ESR) were determined in plasma samples. Inflammatory markers (interleukin 6 (IL-6), IL-10, C-reactive protein (CRP)), NO levels, lipid profile and cortisol levels were measured in serum samples. 

### 2.5. Determination of Thrombosis-Related Parameters

We determined blood-coagulation parameters (fibrinogen levels, prothrombin time and cephaline time) in plasma samples using the BCS XP analyzer (Siemens Healthineers, Erlangen, Germany). We measured determinants of platelet function (platelet count, MPV and PDW), and RBC, neutrophil and lymphocyte counts in plasma samples by flow cytometry using the ADVIA 2120 analyzer (Siemens Healthineers, Erlangen, Germany).

### 2.6. Determination of Inflammatory Markers

The IL-6 level was determined by a chemiluminescent immunoassay using the Access Immunoassay Systems (Beckman Coulter, Pasadena, CA, USA). Levels of IL-10 were measured by a chemiluminescent immunoenzymatic assay using an MLX™ luminometer (Dynex Technologies, Chantilly, VA, USA). CRP was measured using an AU 5800 analyzer (Beckman Coulter). ESR was determined using the SEDI SYSTEM 1 analyzer (Becton Dickinson, New Jersey, USA).

### 2.7. Determination of Lipid Profile

Serum lipid profile (total cholesterol, high-density lipoprotein (HDL)-cholesterol, triglycerides, apolipoprotein A1 and apolipoprotein B) was measured by a spectrophotometric procedure using an OLYMPUS AU 5400 analyzer (Beckman Coulter). Low-density lipoprotein (LDL)-cholesterol levels were estimated indirectly with the Friedewald equation. 

### 2.8. NO Measurement

The reaction of NO with ozone results in the emission of light, and this light, emitted in proportion to the NO concentration, is the basis for the assay that we used in the present study, one of the most accurate NO assays available. NO production was indirectly quantified by measuring nitrate/nitrite and S-nitrose compounds (NOx). The thawed serum aliquots were deproteinized with NaOH 0.8 N and ZnSO4 16% solutions. The total amount of NOx was determined as previously described [18] using the purge system of Sievers Instruments, model NOA 280i (GE Analytical Instruments, Boulder, CO, USA). NOx values refer to the total protein concentrations in the initial samples.

### 2.9. Cortisol Measurement

Cortisol levels were determined in serum samples by a fluorescence polarization immunoassay using an AxSYM analyser (Abbott Laboratories, Illinois, IL, USA). 

### 2.10. Statistical Analysis

The statistical analysis of the data was performed using the statistical package IBM SPSS Statistics 24 for Windows (SPSS Inc, Chicago, IL, USA). The Kolmogorov–Smirnov test and the Levenne test were performed to test normality and homoscedasticity, respectively. To analyze the effects of each olive oil between pre and post measures, the data that followed a normal distribution and the principle of homoscedasticity of variances were tested by paired Student’s t-test to compare differences between means (intra-group comparison). Intra-group analysis of data that did not follow a normal distribution or the principle of homoscedasticity was performed using a Wilcoxon signed-rank test to compare differences between means. To compare the effect of the type of olive oil consumption (EVOO and ROO) in the between-group analysis, all values were converted into delta scores (i.e., post-pre values) and thereafter tested by unpaired Student’s t-test or Mann–Whitney U-test based on normality and homoscedasticity of data. The effect size was calculated using Cohen’s d for parametric tests and the eta squared (η²) for nonparametric tests. Values of Cohen’s d of 0.2, 0.5 and 0.8 correspond to the classical Cohen bands of interpretation of the effect size as small, medium and large, respectively. Values of η² of 0.02, 0.13 and 0.26 correspond to small, medium and large effect size, respectively. We set the level of statistical significance at *p* < 0.05. 

## 3. Results

### 3.1. Participants

Patients diagnosed with FM were randomized into two groups of similar age (mean age of 54.10 ± 5.57 years for the EVOO group and 49.81 ± 5.81 years for the ROO group). All participants consumed all the required olive oil containers, which were returned empty after the end of the nutritional trial. The values of weight and BMI of patients with FM were not significantly modified by any of the olive oils consumed (Table 2). There were statistically significant differences between pre and post measures in the ROO group for waist circumference (*p* < 0.05; Table 2). The consumption of ROO reduced waist circumference in women with FM. Waist circumference showed a medium value for effect size (Table 2).

### 3.2. Effects of Each Olive Oil Type on Laboratory Parameters in Patients with FM

There were statistically significant differences between pre and post measures in the EVOO group for RBC count (*p* < 0.05) and ESR (*p* < 0.05; Table 2). The consumption of EVOO for 3 weeks reduced RBC count and ESR in women with FM. Both parameters showed large values for effect size (Table 2). There were statistically significant differences between pre and post measures in the ROO group for MPV (*p* < 0.05), PDW (*p* < 0.05), NLR (*p* < 0.05), ESR (*p* < 0.05) and fibrinogen (*p* < 0.05; Table 2). Dietary intake of ROO significantly increased MPV, and decreased PDW, NLR, ESR and fibrinogen in patients with FM. All these markers showed large values for effect size (Table 2).

### 3.3. Effects of Type of Olive Oil on Laboratory Parameters in Patients with FM

Statistically significant differences were found in pre–post change between the EVOO and ROO groups for PDW (*p* < 0.05) and cortisol (*p* < 0.05; Table 2). PDW values declined significantly post-treatment in the ROO group in comparison to the EVOO group. Cortisol levels decreased in the EVOO group, while they increased in the ROO group. Both PDW and cortisol showed large values for effect size (Table 2).

## 4. Discussion

The aim of this study was to investigate the effects of two types of olive oil, EVOO and ROO, on cardiovascular risk factors in women with FM.

It has long been known that several cardiovascular diseases are directly related to thrombus formation [19]. Therefore, we analyzed thrombosis-related parameters, such as blood coagulation parameters, platelet indices and RBC count before and after EVOO and ROO intake in women with FM. Our results showed that the consumption of EVOO significantly reduced RBC count in patients with FM. The intake of ROO increased MPV and declined PDW and fibrinogen levels in these patients. Fibrin and platelets are the constituents of arterial thrombi, while venous thrombi are composed of RBCs and fibrin [20]. Fibrin is a fibrillar protein that is formed from fibrinogen by the action of the protease thrombin. PDW and MPV estimate platelet function and their increased values may be considered risk factors for cardiovascular disease [21]. In a previous study, we found altered levels of thrombosis-related parameters, including higher fibrinogen levels, PDW values and RBC counts, and lower MPV values in patients with FM in comparison to controls [7]. Results of the present study show that olive oil may return altered levels compared to those of the healthy controls (data from healthy subjects can be consulted at Molina et al., 2019 [7]). Curiously, although increased MPV values may be considered as a cardiovascular risk factor, our previous data showed significantly lower MPV values in FM patients that may be in a prothrombotic state when compared to healthy subjects [7]. In this regard, it has been reported that MPV is not as specific a marker of platelet activity as PDW because it increases during simple platelet swelling [22]. Moreover, other authors reported finding an inverse relationship between MPV and platelet count [23], as we observed in our previous study [7]. The increase in MPV values due to ROO consumption in the present study would not be negative, since it approximates values of patients with FM to those of healthy subjects [7]. Multiple beneficial effects on cardiovascular disease risk have been associated with the consumption of olive oil, especially of EVOO, including a favorable lipid profile, a reduction of inflammation and the creation of a less prothrombotic environment [8]. Olive oil consumption has been shown to have beneficial effects on platelet aggregation [24] and count [25], as well as on coagulation by reducing tissue factor, factor VII and plasma activator inhibitor type-1 [24]. Few studies have analyzed the levels of fibrinogen after ingestion of olive oil, and the results are not conclusive. Olive oil lowered plasma fibrinogen in women with high baseline fibrinogen levels [16] and in healthy young males [26]. Contrarily, another study did not find differences in fibrinogen levels between olive oil consumers and not consumers [27]. In the present study, EVOO and ROO improved several of the thrombosis parameters analyzed in patients with FM, which may reduce the risk for thrombosis-related cardiovascular disease.

We have also analyzed several inflammatory markers because of their relationships with cardiovascular diseases [28]. Our results showed that the consumption of both EVOO and ROO significantly reduced ESR in women with FM, while ROO also diminished NLR, suggesting that both EVOO and ROO may have antiinflammatory properties in patients diagnosed with FM. ESR is an acute phase reagent; that is, a nonspecific marker whose elevation may involve inflammatory processes. Data by our research group showed elevated ESR values in patients with FM in comparison to healthy subjects [29], and consumption of both olive oils approximates values of patients with FM to those of healthy subjects (data from healthy subjects can be consulted at Ramírez-Tejero et al., 2018 [29]). Several authors did not find significant changes in ESR after consumption of EVOO in comparison to ROO in HIV-infected patients. However, the same authors found that the EVOO intervention reduced ESR compared to baseline values in a subgroup of HIV-infected patients receiving antiretroviral treatment [30]. Although we failed to find significant improvements on other inflammatory parameters such as CRP or IL-6 after 3-week olive oil consumption in women with FM, results of the PREDIMED study showed that 3-month consumption of a Mediterranean diet supplemented with virgin olive oil reduced CRP and IL-6 levels in subjects at high risk for cardiovascular disease when compared with a low-fat diet [31,32]. These different results may be due to the short duration of our trial in comparison to that of the PREDIMED study. However, a short-term nutritional trial, such as the present one, may offer certain advantages with respect to a long-term trial, as it allows patients to adhere to a stricter and more controlled diet in relation to the intake of olive oil and other antioxidants. In this way, the possible interference of other antioxidants can be limited, as can interference from possible changes in the lifestyles of the patients, which could also alter the results [33].

Our data indicate that the antithrombotic and antiinflammatory capacities of both types of organic olive oils in patients diagnosed with FM may not depend on the minor components of olive oil, since ROO is poor in those compounds. Therefore, the beneficial properties of olive oil in women with FM may be related to its fatty acid composition, rich in MUFAs. In fact, the cardioprotective properties of olive oil have been long attributed to its high MUFAs content, mainly oleic acid, which is less susceptible to oxidation than other types of fatty acids. Ingestion of any type of olive oil, EVOO or ROO, increases the plasma oleic acid content of the low-density lipoprotein (LDL) and oleate-rich LDL is less susceptible against oxidation. This is due to the fact that polyunsaturated fatty acids (PUFAs) are key substrates for lipid peroxidation, whose propagation chain is going on via the double bonds of the fatty acid [34]. On the other hand, although a greater beneficial effect on cardiovascular events has been reported for virgin olive oil than for the refined variety [35], the effects were similar for both varieties on overall mortality [36].

Our results have not shown significant changes in another cardiovascular risk marker, the lipid profile, after consumption of any type of olive oil in women with FM. These data agree with a previous work that did not find differences in total cholesterol, LDL-cholesterol, HDL-cholesterol or triglycerides after 4 months of consumption of olive oil in comparison to baseline values in patients with early atherosclerosis [24]. Similarly, another study reported no changes in total cholesterol, LDL-cholesterol or HDL-cholesterol after 3 months of consumption of EVOO in patients with metabolic syndrome in comparison to subjects following their usual diet [37]. However, the same authors found a statistically significant decrease in total cholesterol and LDL-cholesterol after consuming EVOO together with fish oil when compared with their usual diet and the consumption of EVOO alone, respectively [37]. On the other hand, the 3-month consumption of a Mediterranean diet supplemented with virgin olive oil reduced total cholesterol and LDL-cholesterol and increased HDL-cholesterol in comparison to baseline values in high-cardiovascular-risk participants [38]. The same authors in another study with a larger cohort found that LDL-cholesterol decreased and HDL-cholesterol increased in the group of the Mediterranean diet supplemented with virgin olive oil in comparison to a low-fat diet group in high-risk for cardiovascular disease subjects [32]. One reason for our results of unchanged lipid profile may be that the duration of the trial was insufficient to affect the markers analyzed. However, the promising results of the present study should be noted, since we have found that 3 weeks of consumption of olive oil may improve a significant number of cardiovascular risk parameters compared to studies that conducted longer-duration trials.

When analyzing the effects of the type of olive oil on cardiovascular risk markers, significant differences were found in pre–post change between the EVOO and ROO groups for PDW and cortisol. The consumption of EVOO practically did not modify PDW values in women with FM, while the intake of ROO reduced them. Elevated cortisol levels have been proposed as a risk factor for cardiovascular disease [39,40]. The results of the present study have shown that cortisol levels decreased in the EVOO group and increased in the ROO group, evidencing the beneficial effects of EVOO in reducing cortisol levels in patients with FM. Oleic acid, an omega-9 monounsaturated fatty acid, is the main constituent of olive oil. A recent study reported that omega-9 decreased cortisol levels in septic mice [41]. In agreement with our results, a study similar to ours that investigated the effects of the consumption of EVOO in comparison to ROO in HIV-infected patients reported no significant differences in several cardiovascular risk markers (IL-6, fibrinogen, total cholesterol, HDL-cholesterol, triglycerides, ESR) after EVOO intake. Additionally, they did not find any difference after ROO consumption, except for a lower LDL-cholesterol level [30].

The vascular endothelium plays a critical role in the pathophysiology of several cardiovascular diseases. Under oxidative stress conditions, the enhanced production of reactive oxygen species (ROS) alters the vascular tone, which is mediated by the reduced bioavailability of NO, the most potent endogenous vasodilator [10]. The impaired endothelium-dependent vasodilation is the onset of endothelial dysfunction [11]. Our results have not shown significant changes in NO levels after the consumption of any type of olive oil in women with FM. Endothelial NO production derives from endothelial nitric oxide synthase (eNOS). It has been reported that both NO levels and eNOS expression can be modulated by olive oil consumption. Consumption of pomace olive oil enhanced eNOS expression and NO levels in spontaneously hypertensive rats in comparison to animals consuming ROO [42]. The authors suggested that these effects may be associated with a minor component from pomace olive oil, the oleanolic acid, since pomace olive oil has the same concentration of oleic acid as olive oil, but a higher proportion of oleanolic acid [42]. Another study showed that consumption of dark chocolate enriched with EVOO improved blood levels of circulating endothelial progenitor cells, a well-known marker for endothelial function [43].

Finally, we investigated whether olive oil consumption had any effect on the anthropometric characteristics of women with FM. Our results showed that ROO consumption reduced waist circumference in patients with FM. However, patients did not significantly modify weight or BMI after the ingestion of the olive oils. A previous study did not find differences in weight, BMI or waist circumference in asymptomatic high-cardiovascular-risk subjects after 3 months of consumption of a Mediterranean diet supplemented with virgin olive oil [38]. However, a recent meta-analysis reported that diets enriched in olive oil reduced weight, BMI and waist circumference, proposing that it can be an important weight control strategy [44]. The proposal of using olive oil as a weight control strategy may be beneficial for patients with FM, since FM is related to a higher prevalence of overweight and obesity than what occurs in the general population [45].

The main limitation of the present study is the small sample size. However, the parameters that showed statistically significant differences presented large values for effect sizes, indicating that the differences observed after olive oil consumption may be meaningful regardless of sample size. Additional research in a larger sample may be useful to confirm our results.

## 5. Conclusions

It has been reported that patients diagnosed with FM may be at higher risk for cardiovascular disease. Therefore, interventions that could improve cardiovascular risk markers in patients with FM would be desirable. Our results suggest that nutrition may improve markers of cardiovascular risk in these patients. The results of this preliminary study have revealed, for the first time, that consumption of both EVOO and ROO may improve a number of cardiovascular risk markers in patients with FM, evidencing the antithrombotic and antiinflammatory properties of olive oil. These findings recommend the consumption of olive oil as adjuvant for the prevention and/or treatment of cardiovascular diseases in patients with FM.

## Figures and Tables

**Table 1:** Chemical characterization of the olive oils used in the study (EVOO: extra virgin olive oil; ROO: refined olive oil).

COMPOSITION	EVOO	ROO
**Polyunsaturated fatty acids (%)**	4.3	3.8
Linoleic acid (%)	3.9	3.5
Linolenic acid (%)	0.4	0.3
**Monounsaturated fatty acids (%)**	81.7	81.7
Oleic acid (%)	80.7	80.7
Eicosenoic acid (%)	0.3	0.3
Palmitoleic acid (%)	0.7	0.7
**Saturated fatty acids (%)**	14	14.5
Palmitic acid (%)	9.6	10.9
Stearic acid (%)	3.6	2.8
Arachidic acid (%)	0.4	0.4
Behenic acid (%)	0.1	0.1
Others (%)	0.3	0.3
**Tocopherols** (mg/Kg)	203	75
**Polyphenols** (mg/Kg)	248	152

**Table 2:** Anthropometric characteristics and cardiovascular risk markers before and after olive oil consumption in patients with fibromyalgia.

**Anthropometric characteristics**	**EVOO Pre**	**EVOO Post**	***p*^1^**	**Effect Size**	**ROO Pre**	**ROO Post**	***p*^2^**	**Effect Size**	***p*^3^**	**Effect Size**
Weight (kg)	62.57 ± 11.48	62.54 ± 10.85	0.994	0.003	66.59 ± 8.59	66.30 ± 8.83	0.935	0.033	0.709	0.158
Body mass index (kg/m^2^)	25.67 ± 4.71	25.62 ± 4.25	0.928	0.011	26.74 ± 2.24	26.55 ± 2.15	0.852	0.087	0.669	0.188
Waist circumference (cm)	90.32 ± 10.93	87.77 ± 10.28	0.580	0.240	92.88 ± 7.88	88.71 ± 6.84	0.003*	0.560	0.328	0.417
**Blood-coagulation parameters**	**EVOO Pre**	**EVOO Post**	***p*^1^**	**Effect Size**	**ROO Pre**	**ROO Post**	***p*^2^**	**Effect Size**	***p*^3^**	**Effect Size**
Prothrombin time (sec)	11.01 ± 0.40	11.06 ± 0.52	0.812	0.108	11.07 ± 0.59	11.35 ± 0.50	0.243	0.512	0.402	0.372
Cephaline time (sec)	30.16 ± 3.61	30.56 ± 3.15	0.795	0.118	30.54 ± 3.03	30.59 ± 3.11	0.967	0.016	0.689	0.177
Fibrinogen (g/L)	3.71 ± 0.71	3.47 ± 0.51	0.398	0.388	3.20 ± 1.05	2.47 ± 0.34	0.016*^#^	0.935	0.152^#^	0.072
**Platelet indices**	**EVOO Pre**	**EVOO Post**	***p*^1^**	**Effect Size**	**ROO Pre**	**ROO Post**	***p*^2^**	**Effect Size**	***p*^3^**	**Effect Size**
Platelet count (× 10^9^/L)	250.73 ± 85.14	254.09 ± 56.71	0.914	0.046	299.91 ± 66.20	306.64 ± 77.59	0.829	0.093	0.748^#^	0.0009
MPV (fL)	8.25 ± 0.90	8.90 ± 1.65	0.319	0.489	7.55 ± 0.46	8.65 ± 1.02	0.007*	1.39	0.474	0.346
PDW (%)	61.29 ± 12.67	62.25 ± 13.46	0.864	0.073	59.95 ± 11.38	48.41 ± 10.08	0.016*	1.074	0.035*	0.967
	**EVOO Pre**	**EVOO Post**	***p*^1^**	**Effect Size**	**ROO Pre**	**ROO Post**	***p*^2^**	**Effect Size**	***p*^3^**	**Effect Size**
**Red blood cell count** (× 10^12^/L)	5.03 ± 0.26	4.78 ± 0.21	0.001*	1.058	4.87 ± 0.35	4.68 ± 0.40	0.063	0.506	0.610	0.221
**Inflammatory markers**	**EVOO Pre**	**EVOO Post**	***p*^1^**	**Effect Size**	**ROO Pre**	**ROO Post**	***p*^2^**	**Effect Size**	***p*^3^**	**Effect Size**
IL-6 (pg/mL)	2.28 ± 2.35	2.17 ± 0.94	0.505^#^	0.0009	2.26 ± 2.53	2.22 ± 0.97	0.328^#^	0.0001	0.654^#^	0.0002
IL-10 (pg/mL)	1.53 ± 1.14	1.99 ± 0.44	0.235	0.532	1.54 ± 1.47	2.04 ± 0.87	0.325	0.414	0.525^#^	0.0002
CRP (mg/l)	4.15 ± 4.47	2.81 ± 2.32	0.259^#^	0.034	3.21 ± 5.02	1.55 ± 1.86	0.123^#^	0.046	0.898^#^	0.001
ESR (mm)	22.00 ± 10.38	12.91 ± 7.05	0.019*	1.024	16.45 ± 11.14	8.27 ± 3.94	0.028*	0.979	0.699^#^	0.001
NLR	1.82 ± 0.78	1.58 ± 0.51	0.399	0.364	2.51 ± 0.79	1.76 ± 0.45	0.027*	1.167	0.197	0.569
PLR	155.42 ± 43.93	136.07 ± 34.25	0.287	0.491	191.98 ± 42.54	176.24 ± 60.37	0.509	0.301	0.898	0.058
**Lipid profile**	**EVOO Pre**	**EVOO Post**	***p*^1^**	**Effect Size**	**ROO Pre**	**ROO Post**	***p*^2^**	**Effect Size**	***p*^3^**	**Effect Size**
Total cholesterol (mg/dl)	217.11 ± 26.29	235.67 ± 25.95	0.07	0.711	209.70 ± 24.15	219.00 ± 30.38	0.458	0.339	0.455	0.643
HDL-cholesterol (mg/dl)	63.40 ± 9.38	61.20 ± 9.68	0.612	0.231	63.50 ± 16.51	61.80 ± 17.35	0.825	0.1	0.892	0.062
LDL-cholesterol (mg/dl)	134.44 ± 28.53	154.22 ± 25.38	0.07	0.733	128.40 ± 21.53	139.40 ± 27.82	0.336	0.442	0.423	0.372
Triglycerides (mg/dl)	96.50 ± 42.95	102.70 ± 51.37	0.959^#^	0.004	87.00 ± 37.84	86.30 ± 36.79	0.957	0.019	0.690	0.181
Apolipoprotein A1 (mg/dl)	159.59 ± 21.45	155.50 ± 16.43	0.638	0.214	160.10 ± 27.96	151.90 ± 28.42	0.524	0.291	0.532	0.285
Apolipoprotein B (mg/dl)	99.20 ± 11.04	98.70 ± 13.27	0.928	0.041	93.80 ± 14.09	87.10 ± 16.52	0.342	0.463	0.199	0.597
	**EVOO Pre**	**EVOO Post**	***p*^1^**	**Effect Size**	**ROO Pre**	**ROO Post**	***p*^2^**	**Effect Size**	***p*^3^**	**Effect Size**
**NO (μmol/mg protein)**	30.21 ± 21.56	45.48 ± 43.78	0.612^#^	0.443	27.15 ± 20.81	37.84 ± 14.99	0.300	0.589	0.803	0.125
	**EVOO Pre**	**EVOO Post**	***p*^1^**	**Effect Size**	**ROO Pre**	**ROO Post**	***p*^2^**	**Effect Size**	***p*^3^**	**Effect Size**
Cortisol (μg/dl)	11.18 ± 4.14	8.45 ± 4.44	0.152	0.636	10.36 ± 3.75	12.55 ± 3.88	0.067	0.574	0.010*	1.205

Note. Data are expressed as means ± standard deviations. EVOO: patients with FM consuming extra virgin olive oil; ROO: patients with FM consuming refined olive oil; MPV: mean platelet volume; PDW: platelet distribution width; IL: interleukin; CRP: C-reactive protein; ESR: erythrocyte sedimentation rate; NLR: neutrophil-to-lymphocyte ratio; PLR: platelet-to-lymphocyte ratio; HDL: high-density lipoprotein; LDL: low-density lipoprotein; NO: nitric oxide. *p*^1^: *p* -value of comparison between pre and post measures in the EVOO group; *p*^2^: *p* -value of comparison between pre and post measures in the ROO group; *p*^3^: *p* -value of the pre–post differences between EVOO and ROO groups. The statistically significant differences were expressed as * *p* < 0.05. # The degree of statistical significance was established by applying nonparametric tests (the rest of the variables were tested using parametric tests). The effect size was calculated using Cohen´s d for parametric tests and η² for nonparametric tests.
